# Progress in Medicine

**Published:** 1900-02-17

**Authors:** 


### Progress in Medicine.

### DISEASES OF THE RESPIRATORY SYSTEM.

(*Continued from page* 310.)

**Treatment of Pneumonia.**—Mays[14] advises that in lobar pneumonia treatment be directed to reduction of fever, diminution of the volume of blood in the lungs, and support of the nervous system. All three of these indications are met by the application of ice in large, flat, rubber bags to the head and chest. Two or three should be applied in each situation, and kept in position until the temperature falls. Together with this procedure large doses of strychnine should be given both by the mouth and hypodermically. Capsicum, in doses of 10 to 60 minims in water every three or four hours, acts as a valuable diffusible stimulant. For sleeplessness, ¼ gr. of morphine may be given. Cyanosis is best met by venesection or oxygen inhalations. If necessary, rectal feeding must be resorted to. A series of papers on the treatment of pneumonia was recently read before the New York Academy of Medicine.[15] Wells strongly advocated venesection, followed by the hypodermic injection of saline solution. This procedure was, however, unsuitable in the very young, the aged, or the weakly and anæmic. Babcock dealt with pneumonia in the aged, and recommended large doses of strychnine; alcohol and ammonia in moderate doses were beneficial. Hare practised cold sponging with friction, with ice to the precordium and head. Belladonna, in doses of five to 10 minims every four or eight hours, was, in his opinion, more valuable than strychnine or digitalis. In view of the microbic origin of the disease, Smith[16] aims at saturating the blood with some mild antiseptic, preferably salicylate of soda, or carbonate of creosote. Large doses of calomel have a sedative and antipyretic action of short duration. In cases of pneumonia in children, Chapin had met with good results from the use of nitro-glycerine, and Thomson from chloroform in doses of 30 minims. The antiseptic treatment of pneumonia, and, indeed, of all acute broncho-pulmonary diseases, has a strong supporter in Seifert.[17] The drug he uses is creosotal, creosote carbonate, and he claims that a rapid fall of temperature, a lessened duration, and a freedom from relapses and sequelæ follow its administration. The dose for adults is 2½ drachms in 24 hours, and may be taken in two doses, morning and night, in hot sugared milk, in syrup, or in emulsion. This preparation of creosote does not derange the digestive system, but gives increased appetite. The success of the salicylic treatment of pneumonia as described by Sebring (*Medical Record*, April 22, 1899) is no doubt due to its antiseptic power. For the bronchopneumonia of children, Coutts[18] recommends large doses of belladonna, ¼ gr. of the extract every three or four hours, dispensing with all other internal and external medication. The immediate effects are relief of dyspnœa, fall of temperature, shortened duration of disease, and lessened mortality. Comby,[19] dealing with the same disorder, urges the importance of simplicity in treatment, fresh air, cleanliness, and attention to the hygiene of the mouth and nostrils. Fanoni[20] has treated six cases of pneumonia with Pane's antipneumonic serum, and is satisfied as to its efficiency. Ten cubic centimetres are injected hypodermically along the posterior axillary line between the eleventh and twelfth ribs, and this is repeated twice daily as long as the temperature is above 101 deg. No pain and no local reaction follow the injections, nor do there appear to be any constitutional ill effects. The administration is followed by rapid improvement of the general condition and hastening of resolution.

**Asthma.**—Talma[21] attributes spasmodic asthma to spasm of the muscles of the air passages. The whistling ronchus loudest at the end of expiration, so characteristic of asthma, is caused by a stenosis of the larynx and aditus, and this sound is transmitted from one place to another. Spasm of the bronchi does play a minor part. Patients with asthma should practise respiratory gymnastics, calculated to induce slow, deep breathing and quiet, deliberate talking. Bell,[22] on the other hand, regards true asthma as due to an active vaso-dilatation of the pulmonary vessels from direct or indirect stimulation of the vagus fibres. As confirming this supposition he points to the beneficial effect of ergot given in large doses every half-hour until relief is obtained. Similarly the antispasmodics, including chloroform and ether, act by diminishing the spasm of the vaso-dilators, and poultices, amyl-nitrate, nitro-glycerine, by withdrawing blood from the deeper parts. True asthma must be sharply distinguished from the reflex forms, which may be due either to direct or indirect causes. Amongst the direct causes of reflex asthma are: (1) External mechanical and toxic irritation, vapours, dust, &c.; (2) internal mechanical and toxic irritation, gout, rheumatism; (3) pathological conditions of the lungs, tubercle, bronchitis. Indirectly the chief causes are the following, acting through the vagus: Gastric irritation, naso-pharyngeal and aural, cardiac, and central nervous lesions.

**Coryza.**—Ruheman,[23] Chelmonski, and others deny that simple exposure to draughts or low temperature is capable of exciting the condition known to us as "catching cold." Both agree that the disorder has a bacillary origin. Weather changes, and especially absence of sunshine, play a part in predisposing to the disease. Although many cocci and bacilli have been described as associated with coryza none can be credited as pathogenic. An article on this subject appeared in the *Spectator* of January 7th. It pointed out that colds were unknown in the coldest regions, and that Nansen and his crew escaped entirely until they returned to inhabited regions. It is common knowledge that colds may be transmitted from person to person or from persons to animals. The popular theory of cold causation tends to make people over careful and afraid of fresh air and ventilation, thus increasing their susceptibility to cold and its depressing influences. Nassauer[24] recommends the treatment of coryza on antiseptic principles, namely, by vigorously washing out the nostrils with a diluted solution of permanganate of potash. The nose is first cleared by blowing, then irrigated, and finally swabbed with cotton-wool dipped in the same solution. Saturated cotton-wool plugs are allowed to remain in the nose for half an hour after each application.

**Dorsal Percussion.**—Dorsal percussion is of especial value in the diagnosis of diseases of the viscera in the posterior mediastinum. Ewart[25] recommends that Sansom's plexometer be used. The following anatomical points should be noted. At the back the left interlobular septum rises to the height of the third spine, the right to the fourth; the trachea slants to the right, so the bifurcation is outside the lateral line of the vertebral bodies, at the level of the fifth vertebra; the left auricle, separate from the vertebræ by the aorta and œsophagus, lies a little to the right of the spine. The areas of dulness met in dorsal percussion are—interscapular, post-cordial, post-hepatic, and post-splenic.

The interscapular dulness is bilateral and symmetrical; it corresponds to the pulmonary incisures, with their bronchi, blood vessels, &c. The hepatic dulness extending out to the left joins the apex of the cardiac dulness below the angle of the left scapula. The splenic dulness is along the axis of the eleventh rib, and is separated from the kidney dulness by the tip of the twelfth rib. The post-cordial dulness is represented by an elongated triangular figure, with its long axis extending horizontally by three-quarters of its length to the left of the middle line and by about a quarter to the right; vertically it covers from the seventh to the tenth spinous processes. As a resonant note is obtained over the normal dorsal vertebræ, percussion may be used to detect disease of the spinal column. In pericardial effusion there may be a dull quadrilateral patch at the level of the eleventh and twelfth spines; this is not due to the fluid, but is the dulness from liver, pancreas, and other abdominal solids unusually well conducted to the back. The fifth dorsal spine is duller than the others, as at this point the trachea stops, and is replaced by lymphatic glands. In tubercular disease of lung this area of dulness is enlarged. The left auricle can be percussed out from the back. Its dulness corresponds to the eighth and ninth spines, and is placed symmetrically across the middle line.

[14] Merck's Archives, May, 1899. [15] Med. Rec., June 10, 1899. [16] Med. Rec., Nov., 1899. [17] Lancet, Sept. 9, 1899. [18] Brit. Med. Jour., Jan. 28, 1899. [19] Med. Press and Circ., Feb. 15, 1899. [20] New York Med. Jour., Aug. 26, 1899. [21] Berliner Klin. Woch., No. 52. [22] Edin. Med. Jour., Oct., 1899. [23] Ind. Med. Rec., April 26, 1899. [24] Klin. Therap. Mch., Jan., 1899. [25] Lancet, July 29, 1899.

